# Supplementary material for: How to measure earnings surprises: Based on revised market reaction
Source: PLoS One. 2023 Dec 22;18(12):e0296394. doi: 10.1371/journal.pone.0296394 (PMC10745228; doi:10.1371/journal.pone.0296394)
Supplement: S1 Appendix — (DOCX) [file pone.0296394.s001.docx]

Appendix

The test statistics of stock price defined by Jiang and Oomen (2008) and Jiang and Yao (2013):

$$\frac{V_{\left( 0,T \right)}N}{\sqrt{\Omega_{SWV}}}(1-\frac{RV_{N}}{SWV_{N}})^{d}\to N\left( 0,1 \right) （1）$$

at time$[0,T]$,Stock price within the range$\left\{ S_{t_{0}},S_{t_{1}},\cdots,S_{t_{N}} \right\}$, $t_{0}=0$, $t_{N}=T$, then

$RV_{N}=\sum_{i=1}^{N} r_{i}^{2}$ （2）

$SWV_{N}=2\sum_{i=1}^{N} (R_{i}-r_{i})=2\sum_{i=1}^{N} R_{i}-2\ln(S_{T}/S_{0})$ （3）

including:

$r_{t_{i}}=\ln[S_{t_{i}}/S_{t_{i-1}}]$ （4）

$R_{t_{i}}=S_{t_{i}}/S_{t_{i-1}}-1$ （5）

the estimated of $V_{(0,T)}$ is:

$BPV_{N}=\frac{1}{\mu_{1}^{2}}\sum_{i=1}^{N-1} \left| r_{i} \right|\left| r_{i+1} \right|$ （6）

the estimated of $\Omega_{SWV}$ is:

$\hat{\Omega}_{SWV}=\frac{1}{9}\mu_{6}\frac{N^{3}\mu_{6/p}^{-p}}{N-p+1}{\sum_{i=0}^{N-p} \prod_{k=1}^{p} \left| r_{i+k} \right|}^{6/p},p=6$ （7）

including:

$\mu_{p}=E(\left| X \right|^{p})$, $X\sim N(0,1)$ （8）

$\mu_{1}=\sqrt{2/\pi}$，$\mu_{6}=15$ （9）

the statistical test value is calculated in the three-month rolling window, and the specific steps are as follows:

Step 1: Use the daily return of three-month window$\left[ t_{1},t_{N} \right]$ to do the jump test. If the jump test does not reject the zero hypothesis of no jump, then enter the next three-month window to jump test, otherwise record the jump test statistics $JS_{0}$ and continue with Step 2.

Step 2: Use the median of the sample (expressed as $r_{median}$), if the return on day i are replaced, we test the series for jumps $\left\{ r_{t_{1}},\cdots,r_{t_{i-1}},r_{median},r_{t_{i+1}},\cdots,r_{t_{N}} \right\}$, and save the test statistic as $JS_{i},i=1,\cdots,N$.

Step 3: Construct sequence $JS_{0}-JS_{i},i=1,\cdots,N$, if $JS_{0}-JS_{j}$ iss the maximum of the sequence, then the stock price change on day j is identified as a jump.

Step 4: Use the $r_{median}$ replace the stock return which identified as a jump, and return to step 1 until all stock price jumps are detected.

in the identification of the jump, if the test statistic of the jump $JS_{i}>\text{1.65}$, then the point is $jump_{m,t}\text{=1}$, that the stock price has a positive jump; If the test statistic of the jump point $JS_{i}<-\text{1.65}$, then the point is $jump_{m,t}=-1$, that the stock price has a negative jump; test statistics for jump $-\text{1.65<}JS_{i}\text{<1.65}$, then the point is $jump_{m,t}\text{=0}$, that the stock price did not jump.

1. Jiang G J, Oomen R C A. Testing for jumps when asset prices are observed with noise–a “swap variance” approach[J]. Journal of Econometrics, 2008, 144(2): 352-370.
2. Jiang G J, Yao T. Stock price jumps and cross-sectional return predictability[J]. Journal of Financial and Quantitative Analysis, 2013, 48(5): 1519-1544.
